# An improved porcine model of infrarenal abdominal aortic aneurysm

**DOI:** 10.1038/s41598-025-31690-y

**Published:** 2025-12-17

**Authors:** Marta Stei, Per Arkenberg, Theresa Uebing, Agnesa Mazrekaj, Joscha Mulorz, Dennis Mehrkens, Maysam Ahdab, Philipp Barnowski, Gerhard Sengle, Matti Adam, Valerie Lohner, Verena Hoerr, Ali Nahardani, Claudia Schubert-Quecke, Birgitta Mewes, Johannes Lindemeyer, Holger Grüll, Albert Busch, Sebastian Zimmer, Holger Winkels, Hubert Schelzig, Malte Kelm, Georg Nickenig, Stephan Baldus, Markus U. Wagenhäuser, Martin Mollenhauer

**Affiliations:** 1 Heart Center Bonn, Clinic for Internal Medicine II, University Hospital Bonn, Bonn, Germany; 2 Heart Center, Department of Cardiology, Faculty of Medicine and University Hospital Cologne, University of Cologne, Cologne, Germany; 3 Center for Molecular Medicine Cologne (CMMC), University of Cologne, Cologne, Germany; 4 Department of Pediatrics and Adolescent Medicine, Faculty of Medicine and University Hospital Cologne, University of Cologne, Cologne, Germany; 5 Center for Biochemistry, Faculty of Medicine, University Hospital of Cologne, Joseph-Stelzmann-Street 52, 50931 Cologne, Germany; 6 Cologne Excellence Cluster On Cellular Stress Responses in Aging Associated Diseases (CECAD), University of Cologne, Cologne, Germany; 7 Cardiovascular Epidemiology of Aging, Department of Cardiology, Faculty of Medicine and University Hospital Cologne, University of Cologne, Cologne, Germany; 8 Clinic of Radiology, University of Münster, Münster, Germany; 9 Experimental Medicine, University Hospital of Cologne, Cologne, Germany; 10 Core Facility Experimental and Preclinical Imaging Cologne, Faculty of Medicine and University Hospital Cologne, University of Cologne, 50937 Cologne, Germany; 11 Department of Vascular and Endovascular Surgery, Medical Faculty and University Hospital Düsseldorf, Heinrich-Heine-University, Düsseldorf, Germany; 12 Department of Cardiology, Pulmonology and Vascular Medicine, Medical Faculty, Heinrich-Heine-University Düsseldorf, Düsseldorf, Germany; 13 Division of Vascular and Endovascular Surgery, Technical University of Dresden and University Hospital Carl-Gustav Carus, Dresden, Germany; 14 Institute of Molecular Vascular Medicine, TUM Klinikum, Technische Universität München (TUM), Munich, Germany; 15 Experimental Cardiology, Department of Internal Medicine III, Heart Center University Hospital Cologne, LFI Building, Floor 4 R 62, Kerpener Str. 62, 50937 Cologne, Germany

**Keywords:** Abdominal aortic aneurysm (AAA), Surgical porcine model, Structural remodeling, Cytokine profiles, Biomarkers, Cardiology, Diseases, Medical research

## Abstract

Abdominal aortic aneurysm (AAA) remains a significant public health challenge, primarily due to its high mortality rate and the lack of effective preventive and causal strategies. This study aims to establish an improved translational triple-hit porcine model of AAA and to compare it with human AAA disease. AAA was induced in four juvenile domestic pigs by balloon catheter-based aortic dilation, enzyme-mediated extracellular matrix (ECM) degradation, and lysyl oxidase inhibition. The porcine AAA model was characterized by proteomics, histological investigation, and cytokine profiling, and compared with natural occurred human AAA disease. Infrarenal AAA was successfully established with sustained aortic dilation (> 150% of baseline diameter) occurring within 7–14 days post induction and maintained through day 28. Proteomic analysis of porcine AAA tissue identified significant shifts in protein abundance, including downregulation of proteins associated with vascular smooth muscle cell function and ECM integrity, and upregulation of immune-related proteins. Comparative analysis of porcine and human aortic tissues revealed reduced medial elastic fiber content and length, along with increased calcification in porcine AAA, reflecting structural and pathological changes observed in human AAA. Systemic cytokine profiling revealed significant increases in both pro-inflammatory and anti-inflammatory cytokines following AAA induction in pigs, with a cytokine profile largely comparable to that observed in human AAA patients. These findings suggest that the refined porcine AAA model offers a reliable and reproducible platform for investigating AAA progression and evaluating potential therapeutic interventions prior to clinical implementation.

**Supplementary Information:**

The online version contains supplementary material available at 10.1038/s41598-025-31690-y.

## Introduction

Abdominal aortic aneurysm (AAA) is a significant global health concern, with rupture posing an imminent threat to patient life^1^. Annually, AAA accounts for 150,000 deaths worldwide^2^. It is a progressive disease involving various pathomechanisms, such as hemodynamic stress, inflammatory responses, and degradation of the extracellular matrix (ECM), ultimately leading to vessel wall dilation and rupture^3^. Despite ongoing research efforts, no specific pharmacological therapy is currently available, which often leaves surgical intervention as only option once the aneurysm diameter exceeds defined thresholds. To advance our understanding of AAA and develop improved treatment strategies, animal models that closely replicate the human disease are essential. Small animals, particularly rodents, are considered the gold standard in AAA research^4^. Ideally, these models replicate key features of human AAA, such as vascular cell activation, inflammation, intraluminal thrombus (ILT) formation and progressive dilation. However, rodent models often fail to fully capture the complexities of the human cardiovascular system and AAA progression, thereby limiting their translational potential^5,6^. Large animal models, such as pigs, offer significant advantages over small animals as their anatomy and physiology, including genome, fat metabolism, vascular structure, and cardiovascular function, is closer to humans^7^. Existing large animal AAA models often struggle with complications such as vessel ligation, inflammation, and impaired wound healing, potentially affecting experimental outcomes. Refining these models is essential to ensure reliable data and improve animal welfare.

In this context, less invasive techniques and non-invasive monitoring methods such as sonography and MRI offer significant benefits by enabling stable longitudinal data collection, reducing experimental variability, and decreasing the number of animals required. ECM-degrading enzymes and compounds such as elastase, collagenase I, angiotensin II (AngII), and lysyl oxidase (LOX) inhibitors like β-aminopropionitrile (BAPN) play a key role in the development of modern murine and porcine AAA models^8,9^. BAPN, an organic compound found in *lathyrus* plants, has been shown to cause AAA formation and progression in humans^10^, rodents and pigs by inhibiting LOX and disrupting collagen cross-linking^11,12^. In this study, we characterize a modified triple-hit model of AAA in genetically unmodified juvenile domestic pigs and compare its pathophysiological features with human AAA aortas, to enhance the translational relevance of our findings.

## Methods

All methods were performed in accordance with the IUCN Policy Statement on Research Involving Species at Risk of Extinction and the Convention on the Trade in Endangered Species of Wild Fauna and Flora. We confirm that the study was conducted in accordance to the ARRIVE guidelines. Research involving human research participants has been performed in accordance with the Declaration of Helsinki.

### Porcine AAA model

All procedures were approved by the North Rhine-Westphalia Office of Nature, Environment and Consumer Protection (LANUV, Approval number: 81-02.04.2021.A192). Four male castrated Landrace pigs (17 ± 1.4 weeks of age, 62.5 ± 5.8 kg of weight) were included into this study. Pigs were received from Heinrich Genetics, Waldfeucht, Germany. The animals received a daily dose of β-aminopropionitrile (BAPN) at a concentration of 75 mg/kg in lactose-free yogurt from day 7 (d-7) prior until day 28 (d28) after AAA surgery.

### AAA surgery

Anesthesia utilizes a combination of tiletamine, zolazepam (3–5 mg/kg each) and xylazine (1.2–2 mg/kg). After intubation, general anesthesia was maintained with isoflurane (1.5%) and sufentanil (bolus 0.3 μg/kg, infusion 0.25–1.5 ml/kg/h). The pigs received a single intraoperative bolus of cefuroxime (1500 mg in 50 ml saline). Intraoperative monitoring of vital signs involved oxygen saturation, heart rate, electrocardiography, blood pressure, body temperature and electroencephalography. The pigs were positioned in left lateral recumbency, and a flank incision was performed. After dividing the abdominal wall muscles, the peritoneal sac was mobilized from the iliopsoas muscle to expose the aortic segment between the renal arteries and the trifurcation. Retractors were used to stabilize the surgical site. The connective tissue surrounding the infrarenal aorta was carefully removed, and the spinal cord arteries were exposed and marked with vessel loops. Zones caudal to the lower renal artery and cranial to the trifurcation were prepared for aortic cross-clamping and similarly marked. Prior to any intervention on the aortic lumen, 5000 IU of heparin (heparin-natrium, Braun) was administered systemically. A small percutaneous incision distal to the surgical site was made to allow tunneling through the muscles and puncturing the aorta in a flat angle using a biliary needle (Ethicon). Following aortic puncture, a 0.035-inch soft Terumo wire was advanced into the aorta, and a 12F sheath (Cook Medical, Bloomington, IN, USA) was inserted. Correct sheath positioning within the aortic lumen was verified by aspiration. A 16 × 20 mm PTA dilatation catheter (C. R. Bard, Inc., Murray Hill, NJ, USA) was advanced, and the PTA balloon was positioned just outside the sheath within the infrarenal aorta. The balloon was inflated using a pressure syringe and held for 5 min at a constant 2 atmospheric pressure to achieve aortic oversizing. After deflation, allowing for aortic reperfusion, the PTA dilatation catheter was withdrawn from the 12F sheath. Mean arterial pressure (MAP) was maintained above 65 mmHg at all times to ensure adequate perfusion of all end-organs, achieved through the administration of epinephrine (1 ml bolus) and adjustments in intravenous fluid therapy. The Terumo wire was retrieved, lumbar arteries were occluded using bulldog clamps, and aortic cross-clamping was performed. The sheath was flushed with heparinized saline to ensure effective clamping without leakage. A sterile mixture of 250 U porcine pancreas elastase (Sigma-Aldrich) and 4000 U type 1 collagenase (Worthington Biochemical Corp.) in 30 ml saline was prepared prior to surgery. The mixture was infused into the cross-clamped infrarenal aortic segment via the PTA dilatation catheter and immediately aspirated through the sheath to avoid dead space circulation. The mixture was applied under stable pressure for 10 min. Additionally, an enzyme-soaked compress was externally applied to the ballooned segment. Next, the enzyme solution was aspirated, and the sheath was removed. The infrarenal segment was flushed with heparinized saline applied by a blunt, curved needle, and the puncture site was closed using 5–0 Prolene monofilament suture (Ethicon, Raritan, NJ, USA). The bulldog- and aortic clamps were removed. The surgical site was irrigated with saline. The abdominal wall muscles were closed in separate layers using Vicryl 2–0 delayed absorption sutures (Ethicon, Raritan, NJ, USA). Skin closure was performed in Allgower-Donati suture technique (Vicryl 0, Ethicon, Raritan, NJ, USA) and a spray-on bandage was applied for postsurgical wound care. Postoperative analgesia was provided using intravenous buprenorphine at a concentration of 0,01–0,05 mg/kg, administered every six hours for at least five days with a tapered dosing schedule. Animals were sacrificed on day 28 by pentobarbital injection (100 mg/kg).

### Sonography

In the longitudinal view, abdominal aortic diameter measurements were performed 20 mm caudal to the right renal artery on days d-7, d7, d14, d21 and d28 on awake, standing animals that had been pre-trained for the procedure to minimize stress-induced alterations in cardiovascular function. In addition, abdominal aortas were assessed for dissections, hemorrhage, blood turbulences and thrombi.

### Organ collection

Blood samples were taken immediately before and 28 days after AAA induction via a central venous catheter, and plasma was stored at -80 °C. After euthanasia, the retroperitoneum was reopened alongside the surgical scar, and the abdominal aorta was exposed. Both the infrarenal AAA segment and the cranial control (ctrl) segment were carefully dissected and explanted. Aortic samples were rinsed with cooled saline, cut into 1 cm slices, processed for cryo-histology and RNA analysis, and snap-frozen using liquid nitrogen.

### Human samples

The human aortic tissue samples were obtained from the biobank of Department of Vascular and Endovascular Surgery at the Heinrich-Heine-University Düsseldorf (approval number 2018-222_1; study-ID: 2,018,114,854). AAA tissue explants from four patients were included in this study. AAA diameters were measured as follows: patient 1: 5.5 cm; patient 2: 5.6 cm; patient 3: 4.6 cm; and patient 4: 6.0 cm, resulting in a mean maximum diameter of 5.43 ± 0.59 cm. Detailed patient characteristics are provided in Supplemental Table S7. In short, human aortic wall specimens were procured during open surgical AAA procedures. Immediately following excision, the specimens were meticulously washed with a 0.9% NaCl solution and subsequently snap-frozen in liquid nitrogen. Long-term storage was maintained in the gas phase of liquid nitrogen until further analysis. Blood samples were concurrently obtained during the same surgical interventions prior to surgical incision. After centrifugation at 1000xg for 10 min at 4 °C for phase separation, the plasma samples were stored in the gas phase liquid nitrogen for prolonged preservation until subsequent processing.

### Histology

Porcine and human aortic tissue samples were fixated in 4% paraformaldehyde (PFA), dehydrated through a graded ethanol series, cleared in xylene, and embedded in paraffin wax. Tissue sections were cut at a thickness of 4.5 µm for murine samples and 5 µm for porcine and human samples. Sections were stained using Elastica van Gieson, Movat’s Pentachrome, or Von Kossa staining protocols, following the manufacturer’s instructions (Sigma-Aldrich). Immunofluorescence was performed to characterize smooth-muscle cell degradation in porcine aortic aneurysm model tissue and respective controls, as well as human aortic aneurysm samples. Paraffin embedded tissue sections were rehydrated in xylene and decreasing concentrations of ethanol, then washed in PBS for five minutes. Antigen retrieval was subsequently performed in citrate-buffer solution (pH 6) at 100 °C for 10 min. Sections were washed in PBS followed by 10-min permeabilization in 0.1% Triton-X. After another cycle of PBS washing all sections were blocked with Goat-Serum 0.1% for 1 h in humidity chamber at room temperature. Primary antibody (ab5694, Abcam) was diluted in 0.1% Goat-Serum according to species-specific concentration (mouse 1:750, swine 1:500, human 1:500). Antibody-stained sections were incubated at 4 °C in humidity chamber overnight. The Secondary antibody (Goat-anti-mouse R37120, Thermo-Fisher-Scientific), diluted in PBS according to manufacturer’s instruction, was applied on the second day after one cycle of PBS washing. Secondary antibody incubation lasted for 1 h in humidity chamber at room temperature. Following PBS washing sections are stained with DAPI (Sigma-Aldrich) for visualization of nuclei for 10 min and after final PBS washing sealed with Moviol mounting medium. Sections are incubated at 4 °C overnight.

### Microscopy

Histologically stained sections were examined under light microscope (LEICA DM400B). Images for quantification were taken at four different points throughout the stained segment. Staining specific characteristics were analysed using FIJI ImageJ Software according to established protocols which can be provided upon request. All antibody-stained sections were examined under ZEISS fluorescent microscope Axioskop 2 using microscopy camera Axiocam MRm. Images for quantification were acquired again at four distinct measurement-points along each stained section. Analysis for α-SMA positive cells was performed with FIJI ImageJ Software using predefined, species-specific thresholds and an internally established macro.

### Cytokine profiling

Plasma cytokine levels were quantified using species-specific Quantibody® multiplex cytokine arrays (RayBiotech, Inc.): Human Q1 (QAH-CYT-1) and Porcine Q1 (QAP-CYT-1). Samples and standards were processed according to the manufacturer’s protocol. Arrays were incubated with biotinylated detection antibodies and streptavidin-conjugated fluorophores, scanned using a Cy3-compatible laser scanner, and analyzed by densitometry. Cytokine concentrations were calculated based on standard curves.

### Proteomics

Proteomic analysis of porcine abdominal aortic tissue was performed, comparing infrarenal AAA-treated and suprarenal control sections. Tissue homogenization was carried out in Urea lysis buffer (8 M urea with protease inhibitors) using a Precellys 24 Touch homogenizer (Bertin Technologies). Samples were centrifuged, and supernatants further disrupted using a Bioruptor 300 (Diagenode). Protein concentrations were determined with the Pierce™ 660 nm Protein Assay (Thermo Fisher Scientific) after serial dilutions. Equal amounts of protein (50 µg in 50 µl) were reduced with 2.5 µl dithiothreitol (DTT, 100 mM) and alkylated with 5.25 µl chloroacetamide (CAA, 550 mM), followed by dilution in 50 mM TEAB buffer and overnight trypsin digestion (enzyme:substrate ratio 1:75). On the following day, digestion was stopped with formic acid, and peptides were purified using SDB-RP StageTips. StageTips were conditioned with methanol and buffer solutions, peptides were loaded, washed, dried with compressed air, and stored at 4 °C. Mass spectrometric analysis was performed using a Q Exactive Plus Orbitrap LC–MS/MS system (Thermo Fisher Scientific) at the CECAD Proteomics Facility (University of Cologne, Germany).

### Statistical analysis

Data distribution was assessed by Shapiro Wilks Test indicating predominantly normal distribution. Statistical analyses were performed using parametric tests unless differently outlined. Differences between groups were evaluated using student’s t-test, one-way repeated measures analysis of variance (ANOVA) followed by a Tukey post-hoc correction, respectively. Non-parametric analyses were performed with Kruskal–Wallis test followed by Dunn’s post-hoc test. All samples represent biological replicates. Results are expressed as mean ± standard error of the mean (SEM). Statistical analyses were performed using GraphPad Prism 8.4.0 (GraphPad Software, San Diego, CA, USA). Gene ontology (GO) enrichment analysis was visualized with the R package^13^ in R version 4.4.2 [R Core Team, 2020].

## Results

### Establishment of a standardized triple-hit porcine model of AAA

**Fig. 1:**
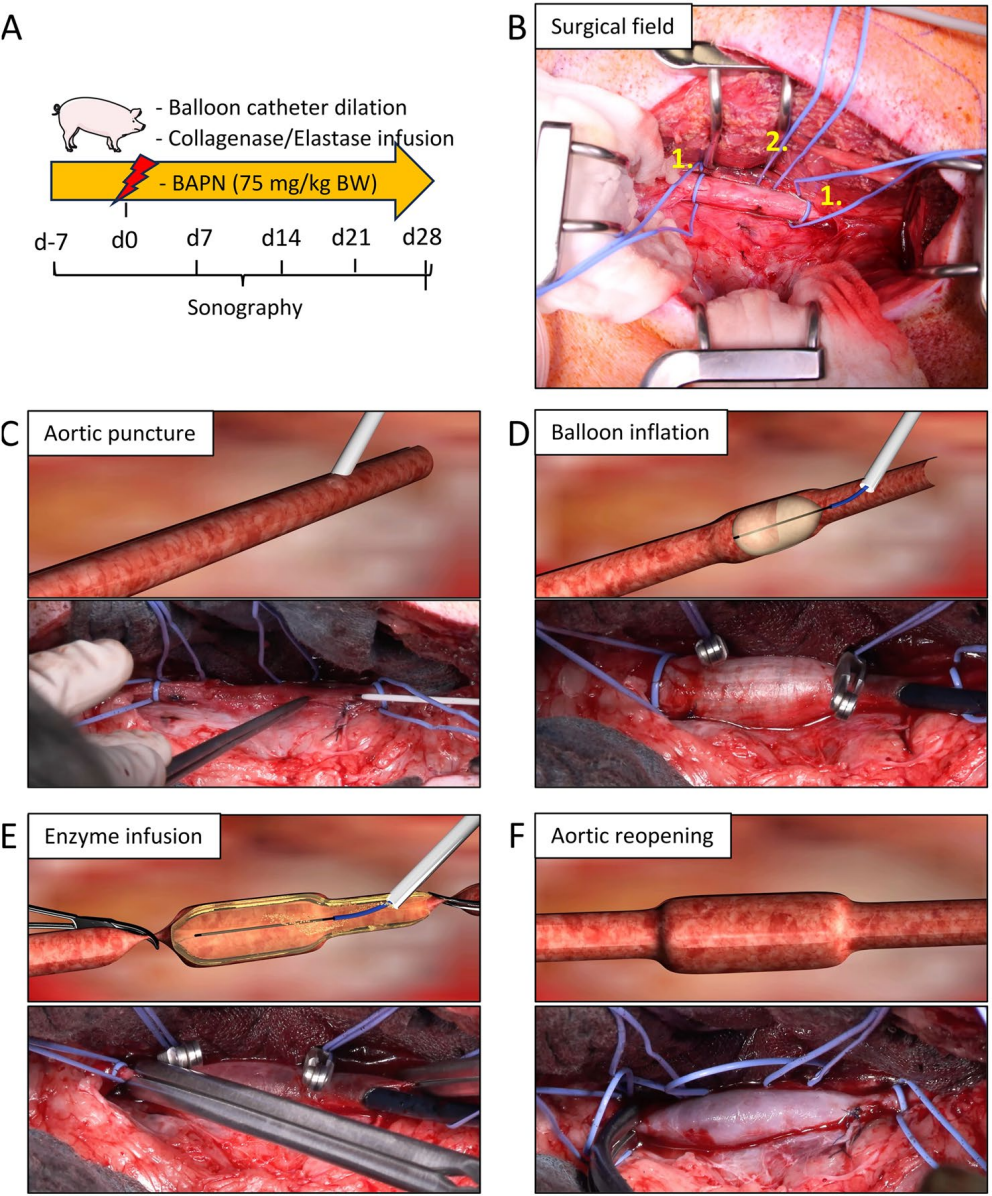
Large animal AAA model. (**A**) Experimental timeline. (**B**) Representative image of the left flank surgical field exposing the infrarenal aorta after aortic reopening. Vessel loops are placed caudal to the renal arteries and cranial to the trifurcation (1.), as well as around the lumbar artery (2.). (**C**) After exposure, the aorta is punctured between the renal arteries and the trifurcation, followed by guidewire-supported insertion of a Cook introducer sheath. (**D**) Balloon catheter insertion followed by balloon inflation at 6 bar for 5 min. (**E**) After catheter removal followed by aortic cross-clamping and occlusion of the lumbar arteries, elastase/collagenase is infused into the dilated aortic segment. (**F**) Upon aortic reopening, restored blood flow reveals a visible dilation of the abdominal aorta.

Four juvenile domestic pigs, aged 16–19 weeks with an average weight of 62.5 ± 5.8 kg, underwent triple-hit surgery to induce AAA. The infrarenal aortic segment was accessed via a retroperitoneal approach (Fig. 1A,B; Supplemental Table S1). The procedure consisted of three sequential interventions: (1) oral administration of BAPN to disrupt ECM integrity, (2) aortic puncture followed by balloon catheter insertion and inflation to induce mechanical stress and endothelial disruption (Fig. 1C,D), and (3) intra-aortic infusion of PPE and collagenase I to enzymatically degrade structural proteins (Fig. 1E). Finally, the aorta was reopened and reperfused (Fig. 1F).

During interventions, no intraoperative complications were observed. Aortic clamping time was below 25 min, and mean arterial pressure remained consistently above 65 mmHg throughout the procedure. Postoperatively, all animals recovered within three to five days, with no major complications such as aortic rupture or hind limb paresis (Supplemental Table S2). Several surgical refinements were implemented, including the use of a retroperitoneal approach and an introducer sheath, to improve standardization and to enhance animal welfare (Supplemental Table S3).

### Stable aneurysm formation following AAA surgery

**Fig. 2:**
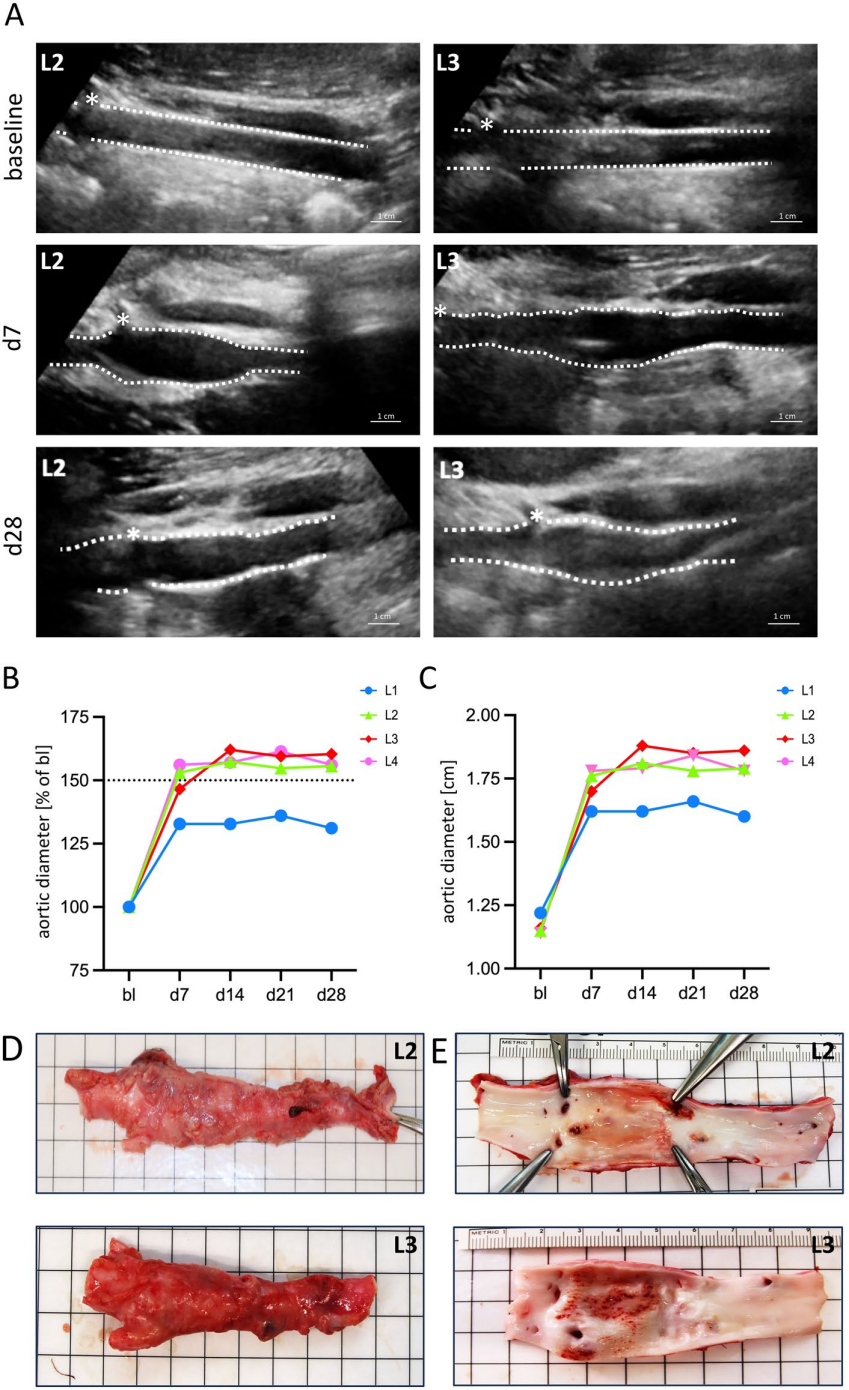
Aortic dilation after AAA surgery. (**A**) Representative sonographic images of the infrarenal aorta at baseline (bl), seven (d7), and 28 (d28) days post AAA surgery. Dashed lines indicate the abdominal aortic wall, *marks the right renal artery. (**B**) Aortic dilation in percent as normalized to baseline (bl) diameter. Baseline diameter was assessed 2 cm distal to the renal artery. (**C**) Respective aortic diameters in cm. Explanted abdominal aortas 28 days after AAA surgery (**D**) before and (**E**) after longitudinal dissection revealing subluminal bleeding and an increase in vessel wall thickness.

Aortic diameter was assessed longitudinally by ultrasound studies before AAA surgery (baseline), with weekly measurements conducted over four weeks post-surgery. Representative images from two animals (L2, L3) show the infrarenal aorta at baseline (bl), day 7 (d7), and day 28 (d28) after AAA induction (Fig. 2A). Aortic dilation reached 152.32 ± 13.22% on day 14 and remained stable at 150.82 ± 13.28% on day 28 compared to baseline (Fig. 2B), which corresponded to an absolute diameter of 1.78 ± 0.11 cm on day 14 and 1.76 ± 0.11 cm on day 28 (Fig. 2C), respectively. A continuous aortic dilation was observed, beginning on day 7 and persisting through day 28 (Supplemental Figure S1, Supplemental Table S1). Accordingly, explanted aortic segments showed pronounced dilation, accompanied by noticeable changes in the adventitia, including inflammatory reddening and fibrotic adhesions to surrounding tissues (Fig. 2D). Macroscopic examination of the luminal surface revealed intra-luminal hematomas and a marked thickening of the aortic wall, consistent with AAA-induced structural remodeling (Fig. 2E). Second harmonic generation (SHG) microscopy performed 28 days after AAA surgery revealed that, in control aortas, densely packed collagen (green) almost completely masks the elastin signal (red). In contrast, AAA aortas showed reduced collagen signal intensity per area and increased interlaminar spacing, making the elastin signal more prominent and visible (Supplemental Figure S2).

### Proteomic signature of porcine abdominal aortic aneurysm

**Fig. 3:**
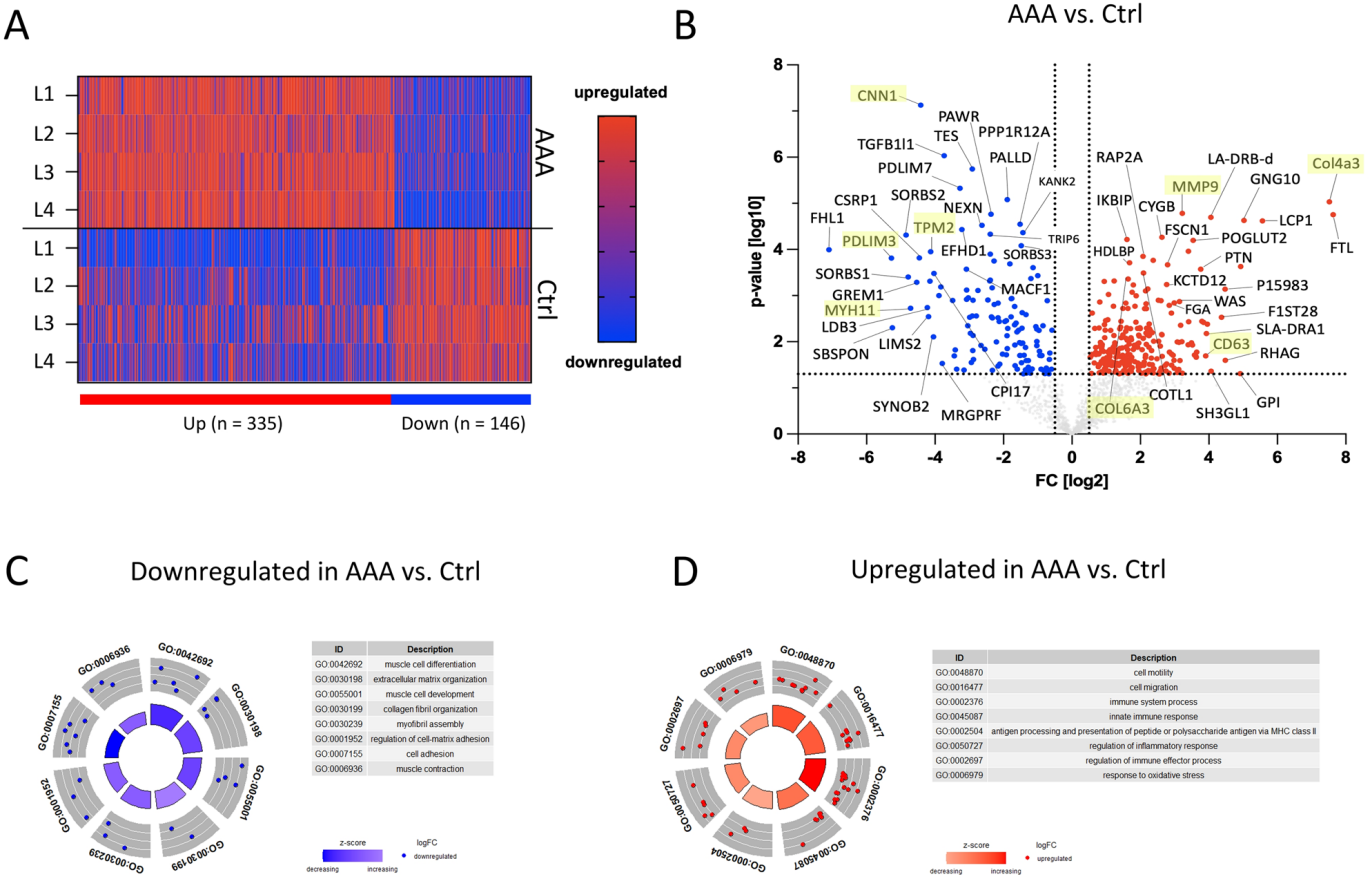
Proteome analysis of porcine AAA. Infrarenal control (ctrl) and AAA porcine aortic segments 28 days after surgery were harvested for bulk proteome analysis. (**A**) Heatmap visualizing the significantly differentially expressed (DE) proteins between ctrl and AAA aortic segments. (**B**) Volcano plot showing the sufficiently up- (red) and downregulated (blue) DE proteins between ctrl and AAA. Important DE proteins are labelled. DE proteins were subjected to pathway analyses. GOcircle plot displays scatter plots of (**C**) down- and (**D**) upregulated proteins in AAA compared to ctrl with their log fold change (logFC) and the most statistically significant GO terms (tables). Inner circle bars indicate the significance of the corresponding GO terms (− log10-adjusted *P*-value), and the color corresponds to the enrichment Z-score calculated by GOplot function in R.

A bulk proteome analysis of porcine AAA vs. control aortic segments 28 days after surgery identified a total of 481 significantly differentially expressed proteins, including 335 downregulated and 146 upregulated (Fig. 3A). Downregulation of *Calponin 1* (CNN1, log2FC = -4.15), *Tropomyosin 2* (TPM2, log2FC = -3.53), *PDZ And LIM Domain 3* (PDLIM3, log2FC = − 4.51) and *Myosin Heavy Chain 11* (MYH11, log2FC = − 4.48) indicates extensive ECM disorganization, degradation, a loss of smooth muscle cells (SMCs) connectivity and structural integrity of the aortic wall (Fig. 3B). Subsequent GO term analysis revealed downregulated genes are highly involved in muscle cell differentiation, cell adhesion, muscle cell development, and ECM organization (Fig. 3C, Supplemental Table S4). Upregulation of *Collagen Type IV Alpha 3 Chain* (COL4A3, log2FC = 7.78) and *Collagen Type VI Alpha 3 Chain* (COL6A3, log2FC = 1.01) indicates an increased amount of ECM proteins within the AAA. Concurrent upregulation of *Matrix Metalloproteinase-9* (MMP9, log2FC = 2.81) suggests extensive ECM remodelling in the presence of a disorganized aortic ECM architecture. Interestingly, *Matrix Metalloproteinase-2* (MMP2, log2FC = 0.84) was not upregulated in AAA. Furthermore, the upregulation of *CD63 Antigen* (CD63, log2FC = 2.69) implies enhanced immune response activity and heightened inflammatory processes within the AAA (Fig. 3B). Subsequent GO term analyses demonstrate that the most upregulated genes were enriched in immune system processes and responses, cell motility, cell migration, response to oxidative stress, and antigen processing and presentation via MHC class II, indicating a dynamic interplay of immune activation, inflammation, and tissue remodeling in the porcine AAA model (Fig. 3D, Supplemental Table S5).

### Analysis of aortic wall morphology and calcification in porcine and human AAA

**Fig. 4:**
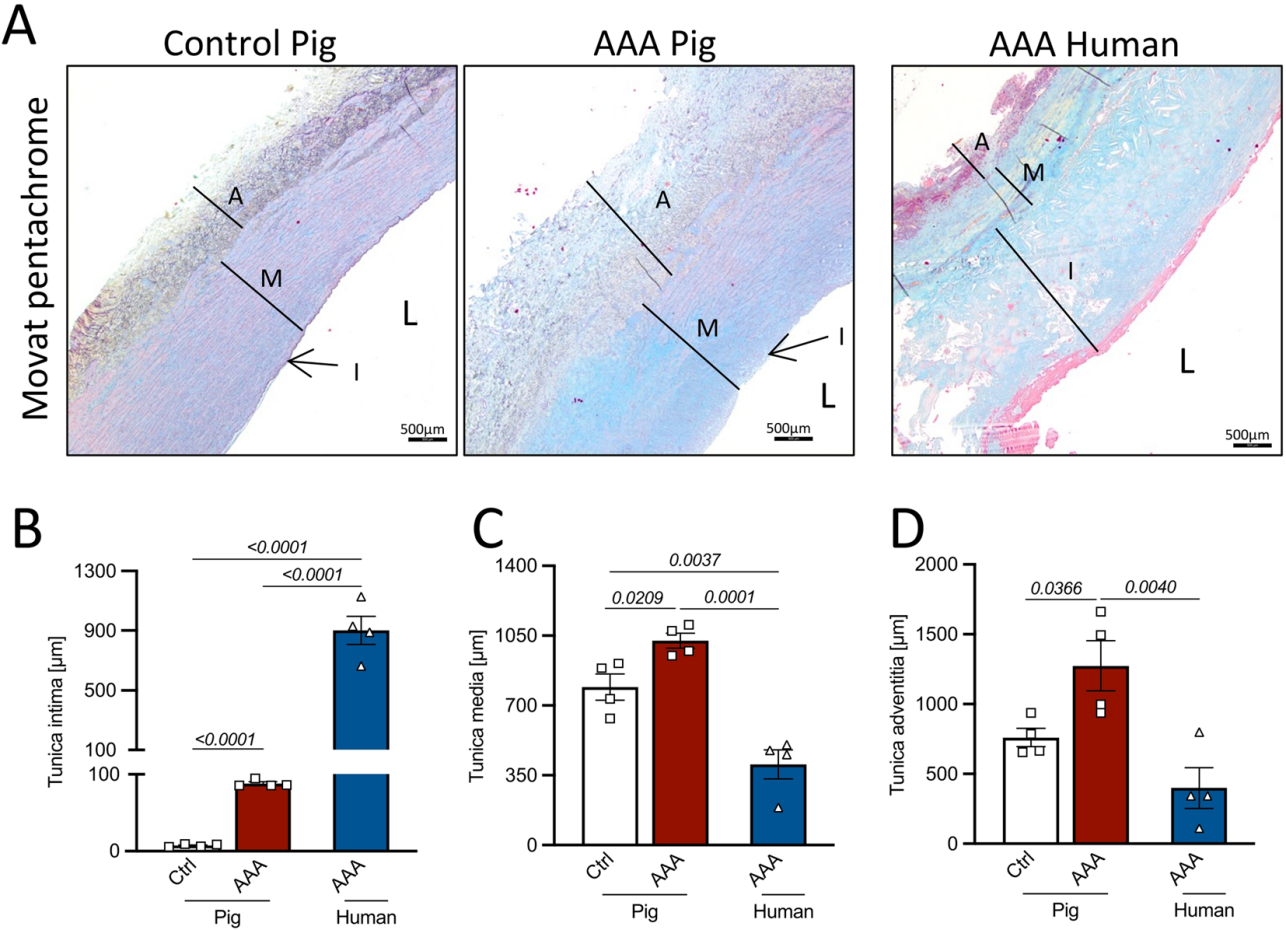
Aortic layer thickness. (**A**) Representative images of Movat pentachrome stainings of suprarenal control and/or infrarenal AAA segments of pig and human aorta. Thickness of the (**B**) tunica intima, (**C**) tunica media and (**D**) tunica adventitia in µm. Normality of data was tested by Shapiro–Wilk test. Significance was determined by student’s t-test for ctrl. vs. AAA (pig) and by one-way ANOVA followed by Tuckey post-hoc test for interspecies AAA data (pig AAA vs. human AAA). Scale bar as indicated. Data is presented as mean ± SEM. *n* = as indicated. L: aortic lumen, I: tunica intima, M: tunica media, A: tunica adventitia.

**Fig. 5:**
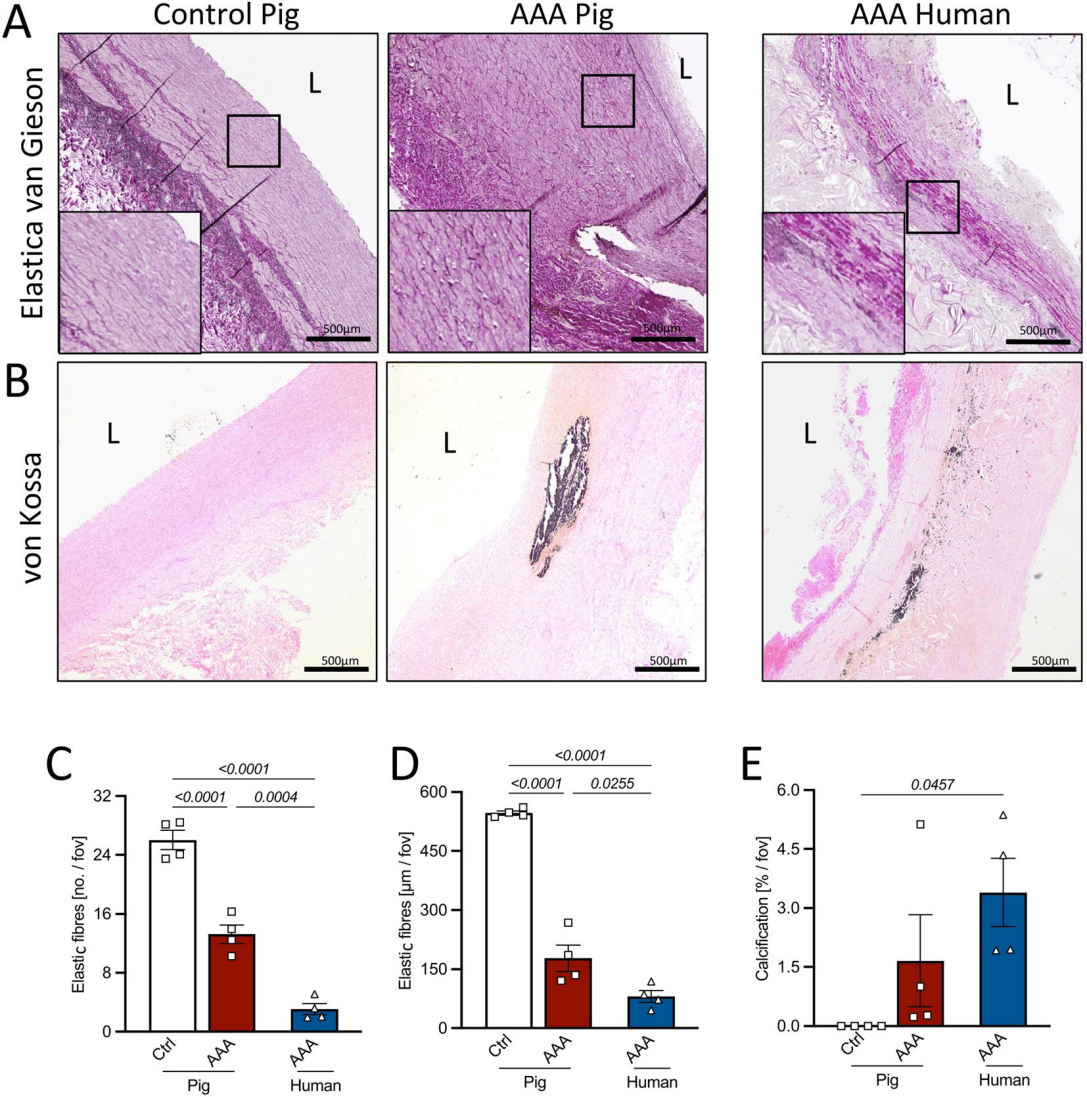
Elastic degradation and aortic calcification. Representative images of (**A**) Elastica van Gieson staining and (**B**) van Kossa staining for calcification of control and/or infrarenal AAA segments of pig and human aorta. (**C**) Number of elastic fibres per field of view, (**D**) length of elastic fibres per field of view and (**E**) amount of aortic calcification within the whole cross-section. Normality of data was tested by Shapiro–Wilk test. Significance was determined by unpaired student’s t-test for ctrl. vs. AAA (pig) and by one-way ANOVA followed by Tuckey post-hoc test for AAA data (pig vs. human). Scale bar as indicated. Data is presented as mean ± SEM. *n* = as indicated. L: aortic lumen. Fov: field of view.

Cross-sections of porcine and human AAA aortas were stained with Movat Pentachrome to assess aortic layer thickness (representative images shown in Fig. 4A). AAA induction in the pigs led to a significant relative thickness increase of all aortic layers (tunica intima, tunica media, and tunica adventitia) (Fig. 4B-D). Compared to human AAA, the porcine intima appeared less prominent (Fig. 4B), while the media and adventitia were thicker both before and after AAA induction (Figs. 4C, D). Elastin degradation and aortic calcification were evaluated by Elastica Van Gieson and Von Kossa staining (representative images shown in Figs. 5A,B). Alpha-smooth muscle actin (αSMA) staining revealed a reduced number of vascular smooth muscle cells (VSMCs) in the tunica media of porcine AAA aortas compared to control aortas, which was comparable to the reduction observed in human AAA samples (Supplementary Fig. 1A,B). Quantitative analysis revealed a significant reduction in the number of medial elastic fibers in porcine AAA compared to control segments. Likewise, the mean elastic fiber length was markedly decreased following AAA induction, mirroring pathological features observed in human AAA (Figs. 5C,D). Of note, aortic calcification was observed in both porcine and human AAA within the medial layer at comparable levels, appearing as nest-like structures in pigs and showing a more circumferential organization in humans (Fig. 5B,E).

### Cytokine profile in porcine and human AAA

**Fig. 6:**
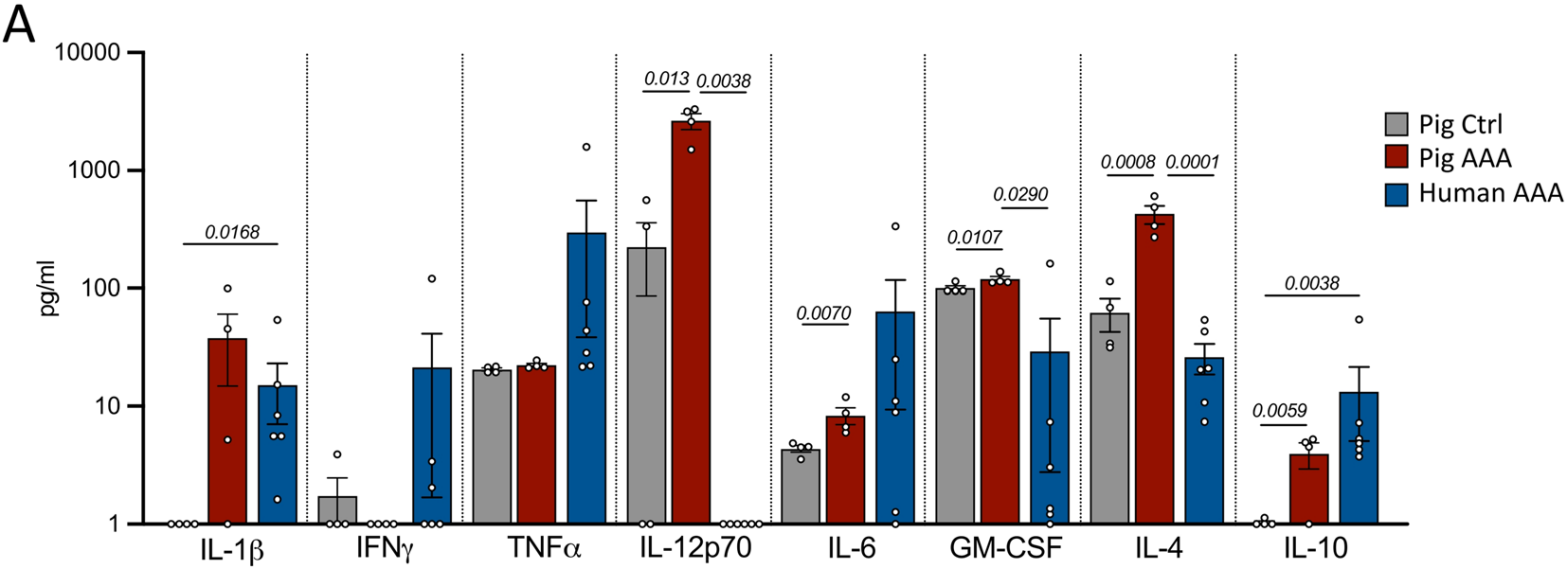
Plasma cytokine levels in AAA. Comparison of plasma cytokines from pig baseline and AAA, and human AAA patients. Normality of data was tested by Shapiro–Wilk test. Significance was determined by unpaired student’s t-test (pig baseline vs. pig AAA) and by Kruskal–Wallis test (for IL-1β, IFNγ, TNFα, IL-12p70, GM-CSF, IL-10) followed by Dunn’s post-hoc test or by ordinary one-way ANOVA (for IL-6, IL-4) followed by Tuckey post-hoc test for interspecies comparisons (pig baseline/pig AAA vs. human AAA).

To assess differences in systemic cytokine levels, plasma collected before and 28 days after AAA induction was analyzed. Following AAA induction pigs showed significantly increased concentrations of inflammatory cytokines, including IL-12p70, IL-6, and GM-CSF, whereas TNFα levels remained unchanged. Interestingly, levels of anti-inflammatory cytokines such as IL-4 and IL-10 were also elevated. Compared to human cytokine profiles, several cytokines including IL-1β, TNFα, IL-6, IL-4, GM-CSF, and IL-10 were consistently detected in both porcine and human AAA plasma. In contrast, IFNγ was undetectable in porcine AAA samples while IL-12p70 was absent from human AAA plasma (Fig. 6A). A comprehensive overview of the cytokine analysis is provided in Supplemental Table S6.

## Discussion

The development of a standalone human-resembling AAA model is challenging due to the complex structure of the aorta and the interplay of pathological mechanisms such as cell death, inflammation, and aortic structural remodeling. Frequently used murine models include the application of AngII via osmotic minipumps^14^, luminal or adventitial perfusion with PPE^15,16^, and adventitial delivery of calcium chloride or -phosphate^17^. None of these models can fully replicate the human pathology. Hallmarks of human AAA, such as ILT formation, intramural hemorrhage, imbalanced proteolysis, angiogenesis, calcification, and aortic rupture, are only present in varying combinations across different models^4^.

In this regard, pigs exhibit striking similarities to humans in terms of genome, fat metabolism, vascular structure, and cardiovascular function, such as resting heart rate and left ventricular pressure^7^, and may therefore better reflect human aortic pathophysiology compared to rodent models. Several porcine AAA models have been developed, utilizing varying combinations of PPE infusion, BAPN treatment, and balloon catheter injury^9,18^, with some studies focusing on aortic rupture^19^ or employing genetically modified *LDLR*^*−/−*^ pigs^20^. Our extended porcine AAA model is based on a previously described triple-hit approach^9,20^ and incorporates key refinements to ensure stable AAA induction and growth, improved surgical handling, and reduced side effects in line with animal welfare: (1) In contrast to the transperitoneal access used in previously described models, the retroperitoneal approach for aortic exposure is associated with markedly reduced surgical trauma, thereby minimizing perioperative stress and inflammation^21^; (2) reduced BAPN dosage to lower lymphocele risk; (3) temporary vertebral artery occlusion to prevent medullary ischemia and paresis; (4) use of a Cook introducer to avoid caudal mesenteric artery ligation and enzyme leakage; (5) testing of aortic clamping tightness with saline before enzyme infusion; (6) pressure-controlled intra-aortic enzyme delivery for standardized inflation; and (7) maintenance of mean arterial pressure > 65 mmHg to ensure organ perfusion during surgery. We observed a maximum relative increase in aortic diameter of approximately 1.5-fold compared to baseline measurements. This corresponds to the threshold at which AAA becomes clinically relevant and when many treatment strategies are specifically designed to intervene during the dynamic phase of diameter expansion—the so-called “window of missed opportunity”^22^.

One key advantage of our established model is its high procedural reliability: All four animals could be successfully operated and examined, resulting in a 100% survival rate and underlining the robustness of the surgical approach. As the procedure does not require highly specialized techniques and is comparatively less complex, the learning curve appears manageable. This makes the model broadly applicable and facilitates its transferability to other research groups.

We compared aortic layer thickness, elastin fiber integrity, calcification, and cytokine profiles between our porcine AAA model and human AAA samples to assess the relevance of these animal models to human AAA pathophysiology. In the context of aortic layer thickening, porcine AAA closely resembles the human condition^23^. Reduced VSMC numbers and elastin fiber degradation and fragmentation were evident in porcine AAA^24^ . Calcification, a known risk factor for aortic rupture in human AAA^25^, was comparable in both species.

Cytokine profiles varied markedly across species: pro-inflammatory cytokines such as IL-1β, GM-CSF, and TNFα were highly expressed in both porcine and human AAA, while IL-12p70 and IL-4 levels were higher in pigs compared to humans. In this context, it is important to consider that although BAPN primarily promotes ECM degradation, its administration may elicit inflammatory responses. While direct interleukin upregulation was low in BAPN-treated mice^26^, other studies have reported IL-3-induced macrophage activation and increased matrix metalloproteinase expression following BAPN supplementation, which also might apply in the pig model^27^.

The proteomic analysis of porcine AAA reveals significant molecular alterations that align with key features of human AAA. The enrichment of proteins involved in muscle cell differentiation, ECM organization, and muscle contraction highlights the impaired function of vascular smooth muscle cells and the compromised integrity of the extracellular matrix. Additionally, the decrease of proteins involved in myofibril assembly further emphasizes loss of contractile function and weakening of vascular stability. Enriched proteins like *CD63*, *MMP9*, and *TRIM28*, associated with immune response, cell motility, and oxidative stress, highlight the role of inflammation and tissue remodeling in porcine AAA progression.

In general, AAA models capture different aspects of the disease, with AngII administered in murine models reflecting persistent pathogenic features such as inflammation and remodeling, while the PPE model (in both rodents and pigs) primarily represents initial wall injury and expansion^28^. Our improved PPE-based approach in pigs should therefore be considered primarily an induction model, although BAPN is administered to ensure progressive wall destabilization and continuous dilatation throughout the experimental period. Establishing a persistent large-animal model remains of great interest but poses substantial challenges, as chronic AngII infusion in pigs is not feasible due to their cardiovascular vulnerability. Future strategies might involve alternative pressor agents or novel systemically applicable ECM-degrading substances, although their potential toxicity would require careful evaluation.

Importantly, the model was established in commercially available Landrace pigs, providing a cost-effective and widely accessible platform for translational AAA research. However, the use of juvenile animals should be taken into account, as structural remodeling processes may differ from those in elderly human patients. In contrast to murine models, the porcine AAA demonstrated pronounced aortic calcification, reflecting a key pathophysiological feature of advanced human disease^29^.

## Conclusion

Given the limited translatability of rodent models into clinical settings^30^, reliable large-animal models are crucial. Our findings indicate that the porcine AAA model presented herein exhibits low variability, meets the clinical criterion of ≥ 150% aortic dilation, and adheres to modern animal welfare standards. Histological analyses reveal clinically relevant tissue and cytokine changes, while complementary proteomic data further underscore the model’s translational value. It offers a promising platform to evaluate novel pharmacologic agents, including small-molecule inhibitors, aimed at slowing or preventing AAA progression prior to clinical translation. Notably, in terms of aortic physiology, it may more closely resemble human AAA, offering a promising platform for preclinical studies on AAA pathophysiology and therapeutic evaluation.

### Limitations

This study has several limitations. Since our data were collected at a single time point, in contrast to the chronic, slow development of human AAA^27^, longitudinal studies would provide more comprehensive insights, particularly into the proinflammatory environment of porcine AAA. Additionally, cytokine profiling across species is complicated due to differences in cytokine structure and assay binding affinities, complicating direct comparisons. Furthermore, the lack of baseline aortic samples from patients prevents us from assessing the progression of aortic dilation and structural changes of human AAA over a defined period.

## Supplementary Information

Supplementary Information 1.

Supplementary Information 2.

Supplementary Information 3.

Supplementary Information 4.

Supplementary Information 5.

## Data Availability

Any additional information required to reanalyze the data reported in this work paper is available from the Lead Contact upon request.
