# Supplementary material for: An improved porcine model of infrarenal abdominal aortic aneurysm
Source: Sci Rep. 2025 Dec 17;15:44059. doi: 10.1038/s41598-025-31690-y (PMC12714784; doi:10.1038/s41598-025-31690-y)
Supplement: Supplementary file 1 — Supplementary Information 1. [file 41598_2025_31690_MOESM1_ESM.pdf]

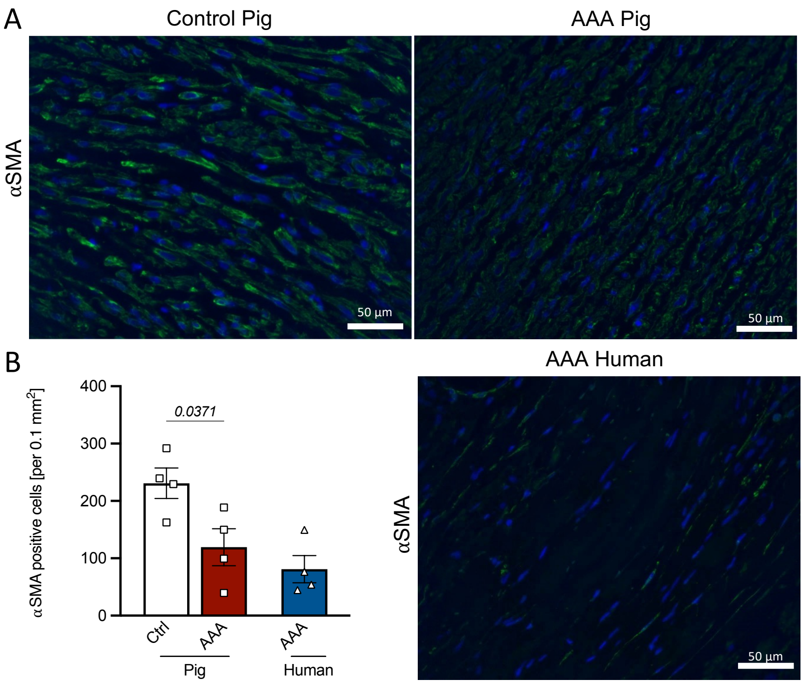

Supplementary Figure 1: Smooth muscle cells in aortic tissue. A) Representative images of αSMA staining in the tunica media of control and infrarenal AAA segments from porcine and human aortas. **B**) Number of αSMA positive cells per 0.1 mm^2 of control and infrarenal AAA segments of pig and human aorta. Normality of data was tested by Shapiro-Wilk test. Significance was determined by unpaired student´s t-test for ctrl. vs. AAA (pig) and by one-way ANOVA followed by Tuckey post-hoc test for AAA data (pig vs. human). Scale bar as indicated. Data is presented as mean ± SEM. *n* = as indicated.
