# Supplementary material for: An improved porcine model of infrarenal abdominal aortic aneurysm
Source: Sci Rep. 2025 Dec 17;15:44059. doi: 10.1038/s41598-025-31690-y (PMC12714784; doi:10.1038/s41598-025-31690-y)
Supplement: Supplementary file 2 — Supplementary Information 2. [file 41598_2025_31690_MOESM2_ESM.pdf]

Supplemental Table S1: Porcine parameters

Timepoint	Parameter	*L1*	*L2*	*L3*	*L4*	*Mean ± SD*
**Baseline**	age (wk)	19	16	16	17	17 ± 1.4
	weight (kg)	65	67	64	54	62.5 ± 5.8
	aortic ⌀ (mm)	12.2	11.5	11.6	11.4	11.7 ± 0.4
**d7**	age (wk)	21	18	18	19	19 ± 1.4
	weight (kg)	70	75	71	66	70.5 ± 3.7
	aortic ⌀ (mm)	12	11.1	11.5	11.8	11.6 ± 0.4
	AAA ⌀ (mm)	16.2	17.6	17.0	17.8	17.15 ± 0.62
**d14**	age (wk)	22	19	19	20	20 ± 1.4
	weight (kg)	76	81	78	70	76.25 ± 4.7
	aortic ⌀ (mm)	12.1	12.1	11.7	12.1	12 ± 0.2
	AAA ⌀ (mm)	16.2	18.1	18.8	17.9	17.75 ± 0,96
**d21**	age (wk)	23	20	20	21	21 ± 1.4
	weight (kg)	82	88	84	78	83 ± 4.2
	aortic ⌀ (mm)	11.8	11.8	12.1	11.9	11.9 ± 0.2
	AAA ⌀ (mm)	16.6	17.8	18.5	18.4	17.83 ± 0.76
**d28**	age (wk)	24	21	21	22	22 ± 1.4
	weight (kg)	86	93	89	83	87.75 ± 4.3
	aortic ⌀ (mm)	11.8	12	11.4	11.8	11.7 ± 0.3
	AAA ⌀ (mm)	16	17.9	18.6	17.8	17.58 ± 0.96

Supplemental Table S2: Intrasurgical parameters

Parameter		L1	L2	L3	L4
**Duration surgery (min)**		164	131	92	125
**Number of clamped lumbal arteries**		2	2	1	1
**Duration aortic clamping (min)**	Balloon inflation	5	5	5	5
	Enzyme installation and flushing	18	20	18	20
**Mean blood pressure (mmHG)**	Pre- aortic clamping	78	74	87	87
	Intra- aortic clamping	-	78	104	98
	Post- aortic clamping	68	72	81	77
**Post-operative femoral pulse quality**		3/5	4/5	5/5	5/5
**Duration of post-operative analgesia (days)**		5	4	4	4

Supplemental Table S3: Key protocol refinements

Non-surgical	Longitudinal non-invasive sonographic assessment of the aortic diameter
	Low BAPN concentration to reduce lymph flow and lymphocele risk
	Stable mean arterial pressure of >65 mmHg to ensure adequate organ perfusion
Surgical	Retroperitoneal access to reduce trauma and post-surgical wound complications
	Temporary vertebral artery occlusion to reduce risk of medullary ischemia and subsequent hind limb paresis
	Introducer sheath usage to avoid ligation of the caudal mesenteric artery and stable enzyme perfusion with standardized pressure without leakage
	Testing the integrity of clamped aortic segments to prevent circulatory instability due to systemic enzyme leakage
	Pressure control for standardized enzyme delivery

Supplemental Table S4: GO-Term analyses of downregulated genes of porcine AAA vs. control aortic segment. n = 4.

Category	Term	PValue	Genes
GOTERM_BP_ALL	GO:0042692~muscle cell differentiation	0.00201603327832613	GREM1, CSRP1, LMOD1, MYH11, MYOZ2
GOTERM_BP_ALL	GO:0030198~extracellular matrix organization	0.0027880608800682142	GREM1, COL18A1, COL4A5, MYH11
GOTERM_BP_ALL	GO:0055001~muscle cell development	0.004599794576628721	CSRP1, LMOD1, MYH11, MYOZ2
GOTERM_BP_ALL	GO:0030199~collagen fibril organization	0.006165415753709966	GREM1, COL18A1
GOTERM_BP_ALL	GO:0030239~myofibril assembly	0.007445510213400201	CSRP1, LMOD1, MYOZ2
GOTERM_BP_ALL	GO:0001952~regulation of cell-matrix adhesion	0.017331497193061807	GREM1, MACF1, DUSP3
GOTERM_BP_ALL	GO:0007155~cell adhesion	0.03622572718973743	ITGA3, TNC, ZYX, CCN3, SORBS1, LIMS2
GOTERM_BP_ALL	GO:0006936~muscle contraction	0.04852835434061774	TPM2, LMOD1, SNTA1

Supplemental Table S5: GO-Term analyses of upregulated genes of porcine AAA vs. control aortic segment. n = 4.

Category	Term	PValue	Genes
GOTERM_BP_ALL	GO:0048870~cell motility	0.0014006551500075311	CD63, TUBA1A, BIN2, SORD, FSCN1, LCP1, PTN, EPX, MMP9, S100A8
GOTERM_BP_ALL	GO:0016477~cell migration	0.0016966368271878589	CD63, TUBA1A, BIN2, FSCN1, LCP1, PTN, EPX, MMP9, S100A8
GOTERM_BP_ALL	GO:0002376~immune system process	0.001754501850108853	GPI, CD74, FGA, KRT1, WAS, PTN, EPX, LA-DRB-D, HPX, TRIM28, CREG1, LCP1, ARL8A, S100A8
GOTERM_BP_ALL	GO:0045087~innate immune response	0.004022246383797096	HPX, CD74, FGA, TRIM28, KRT1, WAS, ARL8A
GOTERM_BP_ALL	GO:0002504~antigen processing and presentation of peptide or polysaccharide antigen via MHC class II	0.007328683184856041	CD74, ARL8A, LA-DRB-D
GOTERM_BP_ALL	GO:0050727~regulation of inflammatory response	0.011797810693736874	CD74, C1QTNF3, KRT1, S100A8, PTGES
GOTERM_BP_ALL	GO:0002697~regulation of immune effector process	0.02069697575472557	HPX, CD74, GPI, WAS, EPX
GOTERM_BP_ALL	GO:0006979~response to oxidative stress	0.037355883250951905	NCF1, GPX8, EPX, MMP9

Supplemental Table S6: Complete plasma cytokine analysis

Species	Pig				Human	
**Group**	**AAA (d28)**		**Ctrl**		**AAA**	
**Parameter**	**Mean**	**SD**	**Mean**	**SD**	**Mean**	**SD**
**IL-1b (pg/ml)**	37.51	45.89	0.00	0.00	22.29	23.00
**IL-4 (pg/ml)**	425.75	149.62	62.36	38.86	38.17	29.13
**IL-6 (pg/ml)**	8.35	2.68	4.36	0.56	94.95	143.77
**IL-10 (pg/ml)**	3.91	2.03	1.03	0.06	21.33	26.08
**GM-CSF (pg/ml)**	119.26	12.30	100.23	9.31	46.20	69.93
**IFNg (pg/ml)**	0.23	0.21	1.73	1.46	42.90	64.43
**TNFa (pg/ml)**	22.10	1.80	20.56	1.20	659.48	982.75
**IL-12p70 (pg/ml)**	2637.32	812.75	223.12	272.43	0.74	0.65
**TGFb1 (pg/ml)**	1200.95	774.76	495.42	743.71	-	-
**IL-8 (pg/ml)**	6.30	0.22	6.26	0.66	-	-
**IL-1a (pg/ml)**	-	-	-	-	18.40	17.66
**IL-2 (pg/ml)**	-	-	-	-	4.67	3.46
**IL-5. (pg/ml)**	-	-	-	-	21.81	24.49
**IL-13 (pg/ml)**	-	-	-	-	5.91	6.95
**MCP-1 (pg/ml)**	-	-	-	-	51.70	26.04
**RANTES (pg/ml)**	-	-	-	-	993.77	733.72
**VEGF (pg/ml)**	-	-	-	-	117.33	164.67
**CXCL1 (pg/ml)**	-	-	-	-	62.06	72.85
**CCL3 (pg/ml)**	-	-	-	-	36.01	78.64
**CCL4 (pg/ml)**	-	-	-	-	24.54	15.70
**MMP-9 (pg/ml)**	-	-	-	-	3892.36	1838.70

Supplemental Table S7: Patient characteristics

Patient No	Age at Surgery	Gender	AAA diameter (cm)	Arterial hypertension	Type 2 diabetes	Coronary artery disease	Obesity	Smoking
1	73	m	5.5	x				x
2	66	m	5.6	x				
3	66	f	4.6				x	x
4	72	m	6.0	x				
